# Host range expansion of Acinetobacter phage vB_Ab4_Hep4 driven by a spontaneous tail tubular mutation

**DOI:** 10.3389/fcimb.2024.1301089

**Published:** 2024-02-16

**Authors:** Penggang He, Feng Cao, Qianyu Qu, Huaixin Geng, Xin Yang, Tong Xu, Rui Wang, Xu Jia, Mao Lu, Peibin Zeng, Guangxin Luan

**Affiliations:** ^1^West China School of Public Health and West China Fourth Hospital, Sichuan University, Chengdu, Sichuan, China; ^2^Chengdu Phagetimes Biotech Co. Ltd, Chengdu, Sichuan, China; ^3^Non-coding RNA and Drug Discovery Key Laboratory of Sichuan Province, Chengdu Medical College, Chengdu, Sichuan, China; ^4^Department of Dermatovenereology, The First Affiliated Hospital of Chengdu Medical College, Chengdu, Sichuan, China

**Keywords:** *Acinetobacter baumannii*, phage, host range expansion, tail tubular protein B, phage resistance, capsule

## Abstract

Bacteriophages (phages) represent promising alternative treatments against multidrug-resistant *Acinetobacter baumannii* (MDRAB) infections. The application of phages as antibacterial agents is limited by their generally narrow host ranges, so changing or expanding the host ranges of phages is beneficial for phage therapy. Multiple studies have identified that phage tail fiber protein mediates the recognition and binding to the host as receptor binding protein in phage infection. However, the tail tubular-dependent host specificity of phages has not been studied well. In this study, we isolated and characterized a novel lytic phage, vB_Ab4_Hep4, specifically infecting MDRAB strains. Meanwhile, we identified a spontaneous mutant of the phage, vB_Ab4_Hep4-M, which revealed an expanded host range compared to the wild-type phage. A single mutation of G to C was detected in the gene encoding the phage tail tubular protein B and thus resulted in an aspartate to histidine change. We further demonstrated that the host range expansion of the phage mutant is driven by the spontaneous mutation of guanine to cytosine using expressed tail tubular protein B. Moreover, we established that the bacterial capsule is the receptor for phage Abp4 and Abp4-M by identifying mutant genes in phage-resistant strains. In conclusion, our study provided a detailed description of phage vB_Ab4_Hep4 and revealed the tail tubular-dependent host specificity in *A. baumannii* phages, which may provide new insights into extending the host ranges of phages by gene-modifying tail tubular proteins.

## Introduction

Phage therapy is considered a promising treatment strategy as an alternative to classic antibiotics for eliminating multidrug-resistant *Acinetobacter baumannii* (MDRAB) clinical infections (Jin et al., 2012; Mardiana et al., 2022; Zhang et al., 2022). Although phage has been demonstrated to be secure and efficacious against MDR bacterial infections (Wright et al., 2009; Schooley et al., 2017), phage resistance of bacteria and narrow lysis spectrums are still major issues that restrict the development of phage therapy (Yehl et al., 2019). Thus, the coevolution relationships between phages and their hosts require further elucidation.

A continuous evolutionary arms race exists between phages and their hosts (Woolhouse et al., 2002; Boon et al., 2020). To survive bacteriophage infection, bacteria can develop resistance by evolving immune-related systems (Rousset and Sorek, 2023) and by altering, disappearing, or masking bacteriophage receptors on the bacterial surface (Boon et al., 2020; Stokar-Avihail et al., 2023). The recognition and binding of phage to the host are mediated by phage receptor binding proteins (RBPs), which have been identified as tail fiber proteins that determine the host specificity (Mardiana et al., 2022). Phages thus require sufficient variability in their RBPs as adaptive mechanisms to cope with these receptor alterations of bacteria, which present as the gene mutations in tail proteins and endow capacity to phages for circumventing the resistance of non-susceptible strains (Buckling and Rainey, 2002; Pal et al., 2007). Host range expansions of phages driven by gene mutations in tail proteins, mainly in tail fiber proteins, have been demonstrated in multiple studies (Le et al., 2013; Botka et al., 2019; Habusha et al., 2019; Boon et al., 2020; Subramanian et al., 2022; Stokar-Avihail et al., 2023).

In this study, we successfully isolated a novel lytic phage vB_Ab4_Hep4 (Abp4) infecting MDRAB strains. We determined the general biological properties of Abp4 and analyzed its genomic characteristics. Furthermore, we identified a phage mutant (MT) vB_Ab4_Hep4-M (Abp4-M), comprehensively investigated and verified the host range expansion of Abp4-M driven by a spontaneous single mutation of G to C in the gene encoding the tail tubular protein B. Interestingly, this mutation site was demonstrated as a hypervariable repeat locus, its high variability enables phage to expand the host range spontaneously. Additionally, the identification of mutant genes in phage-resistant strains revealed that the capsule is a universal host receptor for phage Abp4 and Abp4-M.

## Materials and methods

### Bacteria strains and growth conditions

*A. baumannii* strains used in this study were isolated from Clinical Medical College and the First Affiliated Hospital of Chengdu Medical College (**Table 1**), and the host strain was named Ab4. All strains were identified by 16S rRNA polymerase chain reaction (PCR). The primers used are listed in **Supplementary Table S1**. All culturing was carried out in lysogeny broth (LB) at 37°C with shaking at 200 rpm.

**Table 1:** Host ranges of phages.

Bacterial strain	Phage	
	vB_Ab4_Hep4	vB_Ab4_Hep4-M
Ab4 (host)	✓	✓
Ab170013	×	✓
Ab6	×	×
Ab9	×	×
Ab12	×	×
Ab14	×	×
Ab17	×	×
Ab18	×	×
Ab19	×	×
Ab22	×	×
Ab23	×	×
Ab24	×	×
Ab25	×	×
Ab26	×	×
Ab29	×	×
Ab33	×	×
Ab44	×	×
Ab45	✓	✓
Ab46	✓	✓
Ab56	×	×
Ab82	×	×
Ab100	×	×

All strains above were isolated from the Clinical Medical College and the First Affiliated Hospital of Chengdu Medical College; “✓” as clear lysis; “×” as no lysis.

### Antibiotic resistance testing of host bacteria

In this study, we determined the antibiotic resistance of host Ab4 using the agar dilution method. The minimal inhibitory concentrations (MIC) of 20 antibiotics such as streptomycin, ciprofloxacin, and imipenem against host bacteria were determined and further interpreted according to the guidelines from the Clinical and Laboratory Standards Institute (CLSI, 2020). The antibiotics employed for the drug sensitivity test and their MIC values for host bacteria are shown in **Table 2**.

**Table 2:** Antibiotic resistance of host bacteria Ab4.

Antibiotics	MIC (µg/mL)	Antibiotic resistance	Antibiotics	MIC (µg/mL)	Antibiotic resistance
Tetracycline	1024	R	Gentamicin	>1024	R
Ticarcillin	>1024	R	Amikacin	1024	R
Oxytetracycline	1024	R	Sulfamethoxazole	<0.25	S
Minocycline	32	R	Rifampicin	8	R
Imipenem	32	R	Cefmetazole	>1024	R
Tigecycline	8	R	Chloramphenicol	256	R
Meropenem	64	R	Doxycycline	128	R
Lincomycin	>1024	R	Ciprofloxacin	64	R
Polymyxin	0.5	S	Streptomycin	>1024	R
Levofloxacin	128	R	Cefoperazone-sulbactam	16	S

“S” indicates sensitivity, and “R” indicates resistance.

### Phage isolation and purification

The phage was isolated from a local wastewater station in Chengdu, Sichuan. Phage isolation and purification were performed as described previously (Song et al., 2021), with minor modifications. First, the centrifuged sewage and Ab4 culture were added to the LB liquid medium. The enrichment culture was then incubated overnight at 37°C, centrifuged (12,000 × g, 5 min), and the supernatant was filtered through a 0.22-μm sterile filter. The filtrate was diluted in gradient, mixed with Ab4 in molten semisolid soft agar (0.75% agar), and poured over solidified 1.5% nutrient agar plates. After cooling and solidification, the plates were incubated overnight at 37°C to observe whether there were plaques. The resulting plaques were subjected to three rounds of plaque purification. Purified phages were stored at 4°C in SM buffer (100 mM NaCl, 8 mM MgSO_4_ · 7H_2_O, and 50 mM Tris-HCl at pH 7.5).

### Transmission electron microscopy

Briefly, high-titer phage particles were spotted on carbon-coated copper grids and negatively stained with 2% (wt/vol) phosphotungstic acid. After drying, phages were observed on a Tecnai G2 F20 field emission projection electron microscope (FEI, United States) at 80 kV to obtain morphological information of single-phage particles.

### Thermal and acid-base stabilities

For the thermal stability test, phage suspensions were incubated at 37°C, 40°C, 50°C, 60°C, and 70°C, and sampled at 0 min, 20 min, 40 min, and 60 min for each temperature. Phage titers were determined by the double-layer agar plate method. To determine the acid-base stability, the phages were mixed with normal saline (NS) with a pH of 1-14 and incubated at 37°C for 2 h. Phage titers were determined as described previously. All experiments were performed in triplicate.

### Optimal multiplicity of infection determination

The multiplicity of infection (MOI) represents the ratio of the number of phage particles to the number of host cells in each infection medium. In this experiment, equal volumes of phage and host bacteria were mixed according to different MOI, and the mixture was added to LB liquid medium to incubate at 37°C for 4 h (Peng et al., 2014), then absorbed the supernatant after centrifugation (5,000 × g, 2 min). The phage titer at each MOI was determined using the double-layer agar plate method, and the MOI with the highest phage titer was the optimal MOI. The experiment was performed in triplicate.

### Adsorption experiments

Phage adsorption to host bacteria was performed as described previously (Song et al., 2021), with minor modifications. The host strains were cultured to the logarithmic growth phase and mixed with phage liquid with an MOI of 0.1. The mixture was incubated at 37°C, sampled from time 0 to 10 min with 1-min intervals, 10 to 20 min with 2-min intervals, and centrifuged at 4°C (13,000×g, 1min) immediately. The titer of the supernatant was determined using the method described above, and the phage adsorbed to the host bacteria was calculated based on the titer.

### One-step growth analysis

The host bacteria were cultured to the logarithmic stage, and phage liquid was added at an MOI of 10, incubated at 37°C for 20 min, and centrifuged (13,000×g, 1 min, 4°C). Discarded the supernatant, resuspended the precipitate in liquid LB medium, and repeated centrifugation to remove the unabsorbed phages. The bacterial suspension was adjusted to 10^8^ colony-forming units (CFUs)/mL, mixed with LB liquid medium, and incubated with rotary shaking (200 rpm, 37°C). Aliquots of 100 µL were sampled from 0 to 90 min with 10-min intervals and centrifuged (13,000×g, 1 min, 4°C). The titer of the supernatant was determined using the double-layer agar plate method. The experiment was performed in triplicate.

### Host range determination of phages

The bacteria strains listed in **Table 1** were used for host range analysis using standard spot tests (Kutter, 2009). Briefly, strains were grown overnight in an LB medium. A double agar overlay was made with indicated strains embedded in the top (0.75%) agar layer. 1 µL purified phage suspension containing 10^6^ plaque-forming units (PFUs) was spotted on the agar overlay and the plates were incubated at 37°C overnight. Lysis characteristics were established at the spot where the phage was deposited.

### Phage sequencing and genome analysis

Bacteriophage DNA was extracted from high-titer phage preparations with phenol-chloroform and sodium dodecyl sulfate (SDS) (Lu et al., 2013). DNA samples were sequenced at the Chengdu Phagetimes Biotech Co. Ltd (Chengdu, China) using the Illumina HiSeq platform. The sequencing method was paired-end sequencing (PE), and the length of the reads was 150 bp. Sequencing data were assembled *de novo* using Metaviral SPAdes v.3.15.3 (Antipov et al., 2020). The complete genome of the phage was annotated and analyzed using a variety of bioinformatics tools as described below (Qu et al., 2023). The complete genome of the phage was predicted by Rapid Annotation using the Subsystem Technology (RAST) server (http://rast.nmpdr.org/) (Overbeek et al., 2014). Genome annotations were checked through sequence comparison of protein sequences by the NCBI Non-Redundant Protein Sequences Database of BLASTx_2.13.0 (https://blast.ncbi.nlm.nih.gov/) (Altschul et al., 1997). The circular genome of the phage was visualized using the CGView server database (http://wishart.biology.ualberta.ca/cgview/) (Stothard and Wishart, 2005) and annotated using Artemis Release_18.2.0 (Carver et al., 2012). To analyze the evolutionary relationship between phage Abp4 and other *Acinetobacter* phages (**Supplementary Table S3**), phylogenetic analysis was generated by the MEGA_11.0 software based on the maximum-likelihood (ML) algorithm with 1000 bootstrap replicates (Qu et al., 2023), and the multiple sequence alignment was performed by MUSCLE (Edgar, 2004). Genome comparative analysis was generated using EasyFigure_2.2.3 (Sullivan et al., 2011) software to reveal how dissimilar these phages are in terms of genomic architecture. To analyze the mutation loci of phage Abp4-M, BWA_v0.7.15 (Jo and Koh, 2015) was used to align clean reads of phages that were serially passaged multiple times using Ab4 bacteria as the host to the assembled genome sequence of Abp4. Samtools_v1.5 was applied to sort the aligned BAM file (Danecek et al., 2021). Finally, IGV_2.16.1 (Robinson et al., 2023) software was used for visual analysis.

### Isolation and characterization of the phage mutant

When the host range of phage Abp4 was determined using standard spot tests, purified Abp4 suspensions from different generations were respectively spotted in the middle of a lawn of Ab170013 and incubated overnight at 37°C. We discovered that the characteristic of lysis was established at the spot where the sixth generation Abp4 was deposited, manifested by an incomplete phage plaque. We speculated that this plaque contained phages with spontaneous mutations, enabling them to lyse non-host strain Ab170013 that the phage Abp4 cannot infect. The phage mutant was isolated by directly picking up the single plaque of Ab170013 agar overlay and propagating on Ab170013. The results of phage Abp4 and its host range variant infecting *A. baumannii* strains were verified using standard spot tests. Phage DNA was extracted and sequenced as described previously, and the sequencing data were compared with the Abp4 genome.

### Phage adsorption efficiency assays and infection curves

Phage adsorption efficiency assays were performed on different *A. baumannii* strains according to a previously reported protocol (Song et al., 2021). 1 mL late-exponential-phase culture of each *A. baumannii* strain (1×10^9^ CFU/mL) and 10 µL diluted phage (10^7^ PFU) were mixed and incubated for 20 min at 37°C with shaking and then centrifuging. To determine the amount of unabsorbed phage, the phage titers remaining in the supernatant were evaluated based on an assay of the double-layer plates. The experiment was performed in triplicate and phage adsorption efficiencies were calculated using the equation ([initial titer – residual titer]/initial titer) × 100%.

All strains were cultured as described above. The bacterial suspension (1×10^8^ CFU/mL) or a mixture of bacteria and phage (1×10^7^ PFU/mL) was added to individual wells of a 96-well microtiter plate. Plates were incubated for 16 h at 37°C, and absorbance readings at 600 nm were recorded every hour using BMG SPECTROstar® Nano Plate Reader (Imgen Technologies, Alexandria, VA, USA).

### Protein expression and molecular modeling

Genomic DNA was used as the phage tail tubular protein B gene cloning template via PCR. The primers used are listed in **Supplementary Table S1**. The PCR products were purified and cloned into the pET-42a vector by homologous recombination. Recombinant plasmids were first heat-shocked into *E. coli* DH5α cells and then covered on LB plates containing kanamycin. The monoclones were verified by PCR, and the plasmids were extracted for sequencing. After the sequencing verification, recombinant plasmids were heat-shocked into expression receptor *E. coli* BL21 cells. Expressed glutathione S-transferase (GST)-tagged recombinant phage tail tubular proteins B induced by isopropyl-beta-D-thiogalactopyranoside (IPTG) were purified on the glutathione-agarose columns and then analyzed by sodium dodecyl sulfate-polyacrylamide gel electrophoresis (SDS-PAGE) in an 8% gel (Geng et al., 2023).

The 761-amino-acid-long sequences of WT and MT phage tail tubular protein B (TTPB and TTPBm) were respectively modeled using the RoseTTAFold (https://robetta.bakerlab.org/) algorithm for three-dimensional (3D) structure predictions (Baek et al., 2021; Liang et al., 2022; Camponeschi et al., 2023). The PyMOL_1.8.0.3 software was used to generate the 3D structural models of TTPB and TTPBm and perform the structural alignment of the two protein monomers (Rosignoli and Paiardini, 2022).

### Competitive adsorption assays

100 µL late-exponential-phase culture of each *A. baumannii* strain (1×10^7^ CFU/mL) and 400 µL purified protein solutions (0.55 µg/µL) or an equal volume of normal saline were mixed and incubated for 20 min at 37°C with shaking. Then 10 µL diluted phage (10^6^ PFU/mL) was added into the mixture, incubating for 20 min at 37°C with shaking at 200 rpm. The mixture was centrifuged at 4°C (13,000×g, 5 min) immediately after incubating, and the amount of unabsorbed phage in the supernatant was evaluated based on an assay of the double-layer plates. The experiment was performed in triplicate and the phage adsorption efficiencies were calculated as described previously.

### Screening and genome sequencing for phage-resistant strains

Phages were mixed with WT Ab4 and Ab170013 separately for cultivation with an MOI of 10, and the mixtures were cultured on double-layer soft agar. Plates were incubated overnight at 37°C, and the resulting colonies were picked and saved for further assays. Genomic DNA of Ab4, Ab170013, and phage-resistant mutants were sequenced at Personalbio Technology Co. Ltd (Shanghai, China) using the Illumina NovaSeq platform with a method of paired-end (PE) sequencing. The quality of the raw sequencing reads was evaluated using FastQC_0.12.0 software (https://www.bioinformatics.babraham.ac.uk/projects/fastqc/), and low-quality reads and adapter sequences were trimmed using Fastp_0.23.1 software (Chen et al., 2018) with the option “–adapter_sequence=AGATCGGAAGAGCACACGTCTGAACTCCAGTCA –adapter_sequence_r2=AGATCGGAAGAGCGTCGTGTAGGGAAAGAGTGT –length_required=50 –cut_right -W 5 –cut_right_mean_quality 20 -e 20 –n_base_limit 5 -w 10”. The genomic mapping tool Burrows-Wheeler Aligner (Li and Durbin, 2009) was used for mapping low-divergent sequences to the reference genome of *A. baumanni* strains, the Genome Analyzer Toolkit (GATK) software (McKenna et al., 2010) for the detection of mutations including base substitutions, deletions, and insertions, and the ANNOVAR software (Wang et al., 2010) for variations annotation. As described previously, genome annotations were checked through sequence comparison of protein sequences by the Nucleotide collection (nr/nt) database of tblastn in NCBI (https://blast.ncbi.nlm.nih.gov/Blast.cgi). The DNA mutations of phage-resistant mutants were further validated using PCR and sequencing, and the primers used were listed in the **Supplementary Material** (**Supplementary Table S1**).

### Statistical analysis

All experiments were performed in triplicate. Statistical analysis was performed using OriginPro2017 (Seifert, 2014) software to plot. For phage adsorption efficiency assays and competitive adsorption assays, a normality test based on independent data was performed using the Shapiro-Wilk method, followed by a Levene test to evaluate the homogeneity of variance of the data. Finally, comparisons between two experimental groups or between the experimental group and the control were evaluated for statistical significance using a t-test. Statistical significance was set at p < 0.05. “*” indicates P < 0.05 and “**” indicates P < 0.01.

### Data availability

The genome sequence data of phages have been deposited in the GenBank database (https://www.ncbi.nlm.nih.gov/genbank/). The accession number of WT Abp4 is OP019135, and MT Abp4-M is OR075895.

## Results

### Phage isolation and characterization

The minimal inhibitory concentrations (MIC) of 20 antibiotics against host bacteria were measured using the agar dilution method. As shown in **Table 2**, *A. baumannii* Ab4 was resistant to multiple antibiotics including aminoglycosides (streptomycin, gentamicin, and amikacin), fluoroquinolones (ciprofloxacin and levofloxacin), carbapenems (imipenem and meropenem), Cefmetazole of cephalosporins, and ticarcillin, and thus was considered to be MDRAB (Falagas et al., 2006).

**Figure 1:**
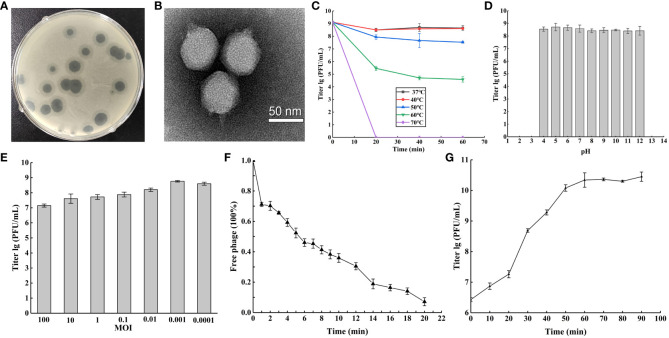
Phage vB_Ab4_Hep4 (Abp4) general biological characteristics. **(A)** Plaques of phage Abp4 on *A. baumannii* Ab4. **(B)** Transmission electron micrograph (TEM) of phage Abp4. Scale bar = 50 nm. **(C)** Tolerance of phage Abp4 to different temperatures. **(D)** Tolerance of phage Abp4 to different pH. **(E)** The optimal multiplicities of infection (MOI) of phage Abp4. **(F)** Adsorption of the phage Abp4. **(G)** One-step growth curve of phage Abp4.

Using MDRAB Ab4 as the host strain, we isolated a previously unidentified phage from sewage water and designated it *Acinetobacter* phage vB_Ab4_Hep4 (referred to as Abp4). Abp4 produced round transparent plaques on the bacterial lawn of Ab4 with translucent halos (**Figure 1A**). Transmission electron microscopy (TEM) showed that the phage had a typical icosahedral structure, with a head diameter of approximately 70 nm, a short conical tail length of approximately 21 nm, and distinct tulle nucleocapsids. So, phage Abp4 has *Podoviridae* morphology (**Figure 1B**).

Thermal and acid-base stabilities are very significant features of a phage, which endow the phage physical tolerance to environmental factors. Abp4 was stable at 37°C to 50°C and weakened above 60°C, and eventually was inactivated at 70°C within 20 min (**Figure 1C**). The results of acid-base stability showed that the phage was stable at pH 4~12 (**Figure 1D**). The optimal MOI of phage Abp4 was 0.001, at this level the phage had the highest production of phage progeny approximately 5.8×10^8^ PFU/mL (**Figure 1E**). The phage adsorption assay showed that more than 90% of phage particles were adsorbed onto the host cells within 20 min (**Figure 1F**). The infection dynamics of the phage were determined by a one-step growth curve, which indicated that Abp4 had a short latent period and reached the plateau stage at 60 min with a burst size of 110 PFU/cell (**Figure 1G**). These results revealed that phage Abp4 maintained high replication capacity and lytic activity.

### Whole-genome characterization and analysis

**Figure 2:**
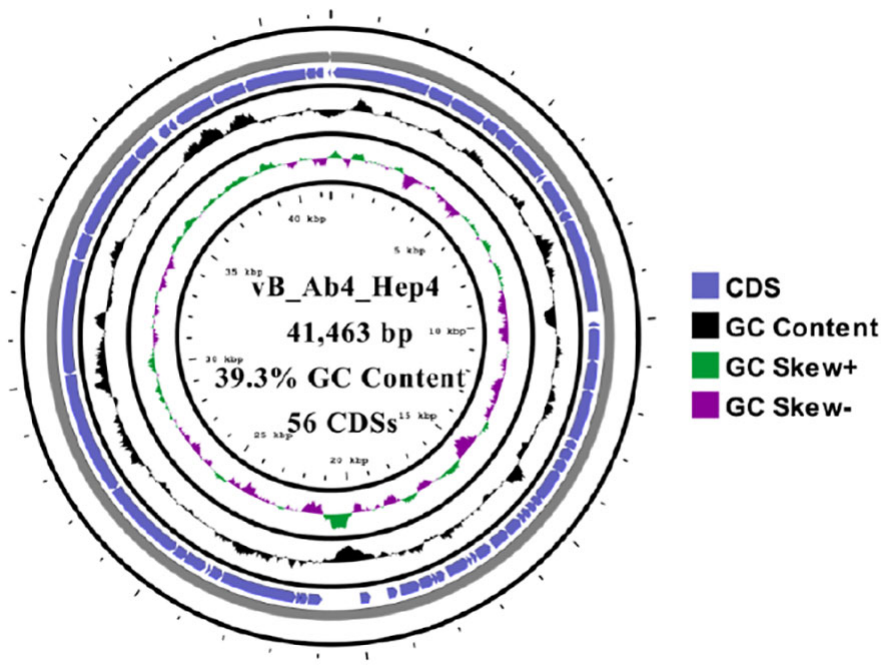
The circular genome map of phage Abp4. Circular genome visualization was performed using the CGView server database.

**Figure 3:**
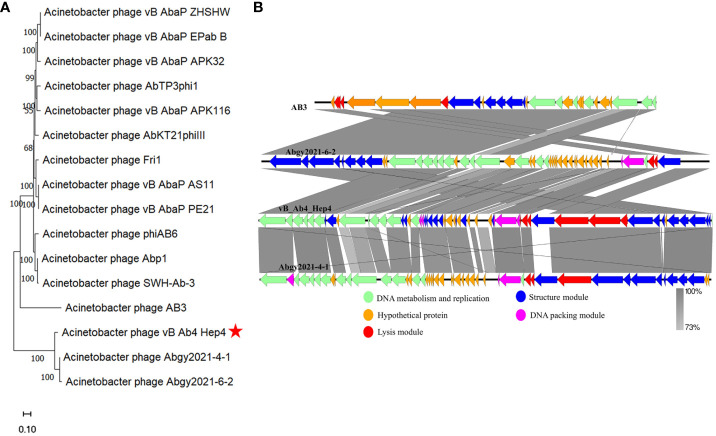
The phylogenetic analysis and comparative genomic analysis between phage Abp4 and other *Acinetobacter* phages. **(A)** The phylogenetic tree with branch length of relatedness between Abp4 and other *Acinetobacter* phages based on whole genome sequences using MEGA_11.0 software. **(B)** The comparative genomic analysis of phage Abp4, AB3, Abgy2021-4-1, and Abgy2021-6-2 was performed based on the results of the phylogenetic analysis. The BLASTn map was performed using EasyFigure_2.2.3. Arrows indicate predicted ORFs, and the arrow’s direction represents the direction of transcription. Different colors denote different functional groups of phage genes.

The whole genome of phage Abp4 was sequenced, analyzed, and deposited in the GenBank database under the accession number OP019135. Whole-genome sequencing results revealed that phage Abp4 has circular double-stranded DNA (dsDNA) with a length of 41,463 bp, GC content of 39.3%, and 56 predicted coding domain sequences (CDSs) (**Figure 2**). The putative functions of the phage proteins were predicted by bioinformatics analysis. All the annotated CDSs can be further categorized into five modules: packing, structure, DNA metabolism and replication, lysis, and other function modules (**Figure 3B**; **Supplementary Table S2**). The structural module comprises 21 ORFs required for phage structural assembly, including Zn-ribbon domain-containing protein, membrane protein, major capsid protein, capsid assembly scaffolding protein, head-to-tail connector protein, tail needle protein, tail fiber protein, and tail tubular protein. The tail fiber protein of phage Abp4 contains 706 amino acids; tail tubular protein A and tail tubular protein B, respectively, consist of 208 and 763 amino acids. None of the genes were found to have functions associated with tRNA, lysogenic, virulence factors, or antibiotic resistance.

To analyze the evolutionary relationship between phage Abp4 and other *Acinetobacter* phages, the original phylogenetic tree with branch length was constructed using the whole genome sequences of phages by the MEGA_11.0 software (**Figure 3A**), and the bootstrap consensus tree was also generated using the same method (**Supplementary Figure S2**). According to the phylogenetic tree and bootstrap values, phage Abp4 clustered with *Acinetobacter* phage AB3 (Zhang et al., 2015a), Abgy2021-4-1, and Abgy2021-6-2 in a single branch in the same genetic subgroup. Based on the results of the phylogenetic analysis, comparative genomic analysis on phage Abp4, AB3, Abgy2021-4-1, and Abgy2021-6-2 located on a single branch in the same genetic subgroup was performed using EasyFigure_2.2.3 software (**Figure 3B**). Phage Abp4 exhibited the high DNA similarity to phage AB3 (95.32% identity), Abgy2021-4-1 (93.86% identity), and Abgy2021-6-2 (93.95% identity) by the genome sequence comparisons in the NCBI nucleotide database. The results of the comparative genomic analysis indicated that the phage Abp4 has the highest functional similarity of putative CDSs to phage Abgy2021-4-1, although it has the highest DNA similarity to phage AB3. Meanwhile, the comparative genomics graph among all phages used for the phylogenetic analysis was also produced to explore how dissimilar these phages are in terms of genomic architecture (**Supplementary Figure S3**).

### Isolation and characterization of the phage mutant

**Figure 4:**
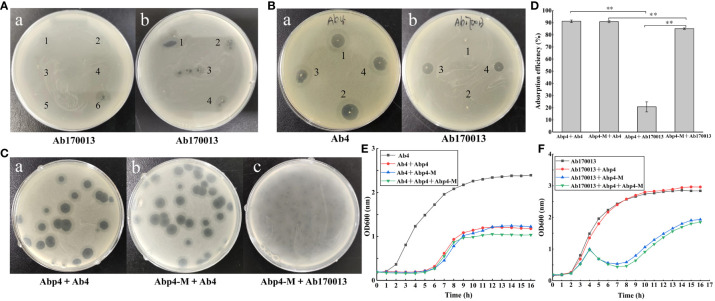
Isolation and characterization of phage mutant vB_Ab4_Hep4-M (Abp4-M). **(A)** Recognition and isolation of phage mutant Abp4-M. (a) The purified suspensions of phage Abp4 from the first generation to the sixth generation were spotted in the middle of a lawn of *A. baumannii* Ab170013, and only the sixth-generation suspension could lyse Ab170013 and form an incomplete phage plaque. (b) The same incomplete phage plaques produced by the sixth-generation phage Abp4 could be observed at all the spots where the phage was deposited. **(B)** The lysis spectrums of wild-type (WT) and mutant (MT) phages to Ab4 and Ab170013. The labels “1, 2” indicate phage plaques produced by Abp4, and “3, 4” represent phage plaques generated by Abp4-M (a, b). **(C)** Plaques of WT and MT phages on Ab4 and Ab170013. **(D)** Adsorption efficiencies of WT and MT phages binding to Ab4 and Ab170013. * P < 0.05 and ** P < 0.01 indicated a significant difference between the two experimental groups. Statistical analysis was performed using a t-test. **(E)** Infection curves of WT and MT phages to Ab4. **(F)** Infection curves of WT and MT phages to Ab170013.

When determining the host range of phage Abp4 by standard spot tests, we accidentally found that the sixth-generation suspensions of Abp4 could lyse *A. baumannii* Ab170013 and form an incomplete phage plaque on the bacterial lawn **Figure 4A**a). This phage suspension was repeatedly spotted in the middle of a lawn of Ab170013 and the same incomplete phage plaques could be observed at all the spots where the phage was deposited **Figure 4A**b). Based on the phenomenon, we hypothesized this phage Abp4 suspension contained phages with spontaneous mutations, enabling them to lyse Ab170013 that could not be recognized by parental phage. Using Ab170013 as an indicator strain, the phage plaques on the Ab170013 bacterial lawn were subjected to three rounds of plaque purification. The purified phage was designated as *Acinetobacter* phage vB_Ab4_Hep4-M (referred to as Abp4-M).

Both phage Abp4 and Abp4-M were spotted on bacterial lawns of Ab4 and Ab170013, respectively, the results demonstrated that phage Abp4-M showed a broader host range and could lyse both Ab4 and Ab170013, while Abp4 could only infect Ab4 (**Figure 4B**). Phage Abp4-M produced round transparent plaques with translucent halos on Ab4 double-layer agar plates, similar to the plaques formed by Abp4 (**Figure 4C**b). However, Abp4-M could only form small and turbid phage plaques on Ab170013 bacterial lawns, indicating lower lysis efficiency (**Figure 4C**c). This observation suggests that the mutation enabling the phage to expand its host range may have a fitness cost (Stokar-Avihail et al., 2023).

### Phage host range, adsorption efficiency assays, and infection curves

As shown in **Table 1**, the host ranges of phage Abp4 and Abp4-M were assessed by standard spot tests against a total of 22 clinically isolated *A. baumannii* strains. The results showed that Abp4 and Abp4-M could also lyse Ab45 and Ab46 besides the host Ab4, producing clear plaques on bacterial lawns. Additionally, phage Abp4-M obtained the ability to lyse Ab170013 and thus appeared to have a broader host range than Abp4. In general, the lysis spectrums of phage Abp4 and Abp4-M we have determined in this experiment were narrow based on the limited number of strains.

To assess the potential influence of mutation on phage adsorption, the adsorption efficiencies of the phages were evaluated (**Figure 4D**). The results showed that both phage Abp4 and Abp4-M exhibited identical adsorption efficiencies, exceeding 90% within 20 minutes, when interacting with host Ab4. However, Abp4 showed minimal adsorption to Ab170013. Meanwhile, Abp4-M had lower adsorption efficiency to Ab170013 than Ab4, which may explain the smaller and turbid plaques observed when Abp4-M lysed Ab170013. The infection curves demonstrated that both Abp4 and Abp4-M could effectively inhibit the growth of Ab4 (**Figure 4E**), and only Abp4-M could efficiently repress the proliferation of Ab170013 (**Figure 4F**). Notably, the infection curve for the Ab4 strain in the presence of a combination of phages Abp4 and Abp4-M was identical to that observed when each phage was used alone, suggesting that Abp4 and Abp4-M are homologous. Ab4 and Ab170013 exhibited different growth curves when infected with the same MOI of Abp4-M, indicating that Abp4-M had different lytic efficiencies against the two bacterial strains (**Figures 4E, F**).

### The mechanism for host range expansion of the phage mutant

**Figure 5:**
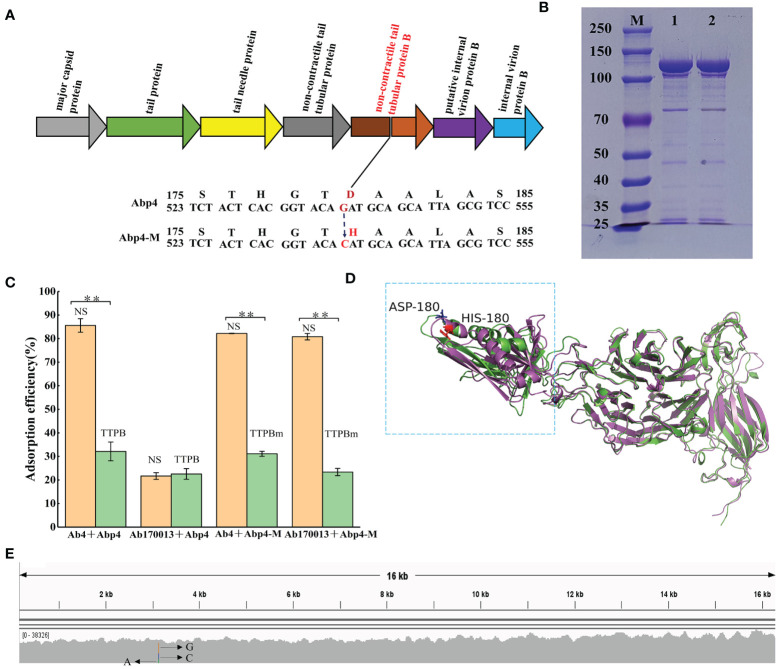
The mechanism for host range expansion of the phage mutant Abp4-M. **(A)** The spontaneous single mutation of G to C in the gene encoding tail tubular protein B of the phage resulted in an aspartate to histidine change. **(B)** Expressed glutathione S-transferase (GST)-tagged tail tubular proteins B of phages were purified on glutathione-agarose columns and then analyzed by SDS-PAGE in an 8% gel. “M” indicates the protein marker. “1” indicates TTPB and “2” represents TTPBm. **(C)** Competitive adsorption assays of phages. As shown in the histogram, green columns indicate the experimental groups added TTPB or TTPBm, and orange columns represent the control groups in which tail tubular protein B was replaced with an equal volume of normal saline (NS). * P < 0.05 and ** P < 0.01 indicated a significant difference between the experimental and control groups. Statistical analysis was performed using a t-test. **(D)** The structural alignment of TTPB and TTPBm monomers. The 3D structure models were predicted by the RoseTTAFold and the structural alignment was generated by PyMOL software. The green monomer indicates TTPB and the magenta monomer is TTPBm. **(E)** The hypervariable mutation locus in the gene encoding tail tubular protein B of the phage, endowed the phage with sufficient variability to expand its host range through spontaneous mutations.

To explore the molecular basis of the phage Abp4-M host range expansion, the genome of Abp4-M was sequenced as described previously, and the sequence data was deposited in the GenBank database under the accession number OR075895. The comparative analysis of genome sequencing revealed that there was a single mutation of G to C at position 21,623 in the phage genome, which resulted in an aspartate to histidine change in the tail tubular protein B (TTPB) of the phage (**Figure 5A**; **Supplementary Figure S1**). Based on the essential roles of phage tail proteins for the recognition and binding of the host cells, we speculated that the host range expansion of Abp4-M may be driven by the spontaneous tail tubular mutation.

Competitive adsorption assays were conducted using recombinant phage TTPB to verify this hypothesis. The recombinant TTPB was purified on glutathione-agarose columns and then analyzed using SDS-PAGE in an 8% gel. Both TTPB and TTPBm exhibited specific protein bandings consistent with the expected size of 110 kDa (comprising tail tubular protein B of 84 kDa and a GST label of 26 kDa) (**Figure 5B**). Competitive adsorption assays showed that both TTPB and TTPBm competitively binding to Ab4 result in a significant decrease in the adsorption efficiency of Abp4 to Ab4. The adsorption efficiency of Abp4-M to Ab170013 was decreased significantly due to the competitive adsorption of TTPBm. On the other hand, the adsorption of Abp4 to Ab170013 was highly inefficient and unaffected by TTPB (**Figure 5C**). These findings were consistent with the phage lysis spectra, indicating that the single mutation of G to C, resulting in the change of aspartate to histidine in the phage tail tubular protein B, was indeed responsible for the host range expansion of Abp4-M.

The comparative results of RoseTTAFold structure prediction showed that the substitution of aspartate with histidine at the 180 amino acid position extended the length of the alpha helix, thereby altering the overall structural configuration of the region spanning from the amino acid residues 140-296 (**Figure 5D**), which was regarded as one of the important reasons for different lysis spectra of phage Abp4 and Abp4-M. As described previously, we used multiple bioinformatics tools including BWA_v0.7.15, samtools_v1.5, and IGV_2.16.1 to analyze the variability of the phage mutation loci. The result indicated that rather than being scattered evenly along the genome, mutations only occurred in this specific site, which exhibits high variability (A accounts for 22%, C 29%, and G 49%) (**Figure 5E**).

### Identification of the receptor for phages

Phage Abp4-M acquired the capacity to infect non-host strain Ab170013 due to the spontaneous point mutation in the tail tubular. We are interested in whether Abp4 and Abp4-M target the same host receptor in Ab4 and Ab170013. To determine the host receptor of phages, the genomes of WT *A. baumannii* strains and phage-resistant mutants were sequenced and comparatively analyzed to identify mutations responsible for conferring phage resistance. The results revealed that the mutant genes of phage-resistant strains randomly selected from different bacterium-phage cocultured plates were all located in capsule biosynthesis gene clusters, suggesting that capsule is the universal host receptor for both phage Abp4 and Abp4-M (**Table 3**).

**Table 3:** Identification of mutations in phage-resistant strains.

Mutants screening	Phage-resistant strain	Mutant type	Mutant gene	Gene product	Putative gene function
Abp4+Ab4	Abp4_4R1	transposon insertion	*pgm* 758 IS5 family transposase (1049bp) insertion	PMM	capsule biosynthesis
	Abp4_4R3		*gpi* 748 IS5 family transposase (1049bp) insertion	G6PI	
	Abp4_4R6				
	Abp4_4R8				
Abp4-M+Ab4	Abp4M_4R1				
	Abp4M_4R4				
	Abp4M_4R6		*ugd* 1,137 IS5 family transposase (1049bp) insertion	Ugd	
Abp4-M+Ab170013	Abp4M_13R4	transversion mutation	*gtrOC20* (T351A)	GT (S224C)	

PMM, phosphomannomutase; G6PI, glucose-6-phosphate isomerase; Ugd, UDP-glucose 6-dehydrogenase; GT, glycosyltransferase.

Among the mutants that come from the cocultured plate of Abp4 and Ab4, *pgm* (encoding a phosphomannomutase, PMM) gene was interrupted by a 1,049-bp ISAha2 transposon of the IS5 family at position 758 in Abp4_4R1, while *gpi* gene (encoding a glucose-6-phosphate isomerase, G6PI) was interrupted by an identical insertion sequence at position 748 in Abp4_4R3, Abp4_4R6, and Abp4_4R8. Both genes, *pgm* and *gpi*, are associated with the capsular polysaccharide (CPS) biosynthesis (Zhang et al., 2015b; Sarkar et al., 2019; McFarland et al., 2021). The phage-resistant mutants Abp4M_4R1 and Abp4M_4R4 exhibited the same transposon insertion as described above in the *gpi* gene. In Abp4M_4R6, another CPS-associated gene *ugd* (encoding a UDP-glucose 6-dehydrogenase, Ugd) was interrupted by ISAha2 at position 1,137 (Cahill et al., 2020). In addition, the *gtrOC20* gene related to CPS biosynthesis (encoding a glycosyltransferase, GT) acquired a T351A transversion mutation and thus resulted in a S224C amino acid substitution in Abp4M_13R4, which originated from the adaptive evolution of Ab170013 to Abp4-M. GT plays an integral role in the capsule surface assembly of bacterial strains. Phage resistance caused by GT gene mutation has been reported in *A. baumannii*, and *Klebsiella pneumoniae* (Cai et al., 2019; Timoshina et al., 2023).

## Discussion

Viral protein destabilizing mutations provide structural flexibility to protein and alter the binding surface’s chemical properties, enabling interactions with the new receptor. This can improve the host range evolvability of phages (Strobel et al., 2022). The first step in phage infection is recognition and binding to the host. As one of the RBPs, tail proteins are essential for host recognition and attachment and further determine the host specificity of phages (de Leeuw et al., 2020; Mardiana et al., 2022). Phage tail fiber-dependent host specificity has been verified in multiple previous studies. A spontaneous mutant phage PaP1 exhibited a broader host range when there was a single-point mutation in the putative tail fiber gene compared with the parental phage JG004 (Le et al., 2013). The host range expansion of *Pseudomonas* virus LUZ7 was also validated to be driven by a conserved tail fiber mutation (Boon et al., 2020). In *B. subtilis* phage SBSphiJ and *E. coli* phage T7, the tail fiber mutations allow phage mutants to escape bacterial defense systems compared with WT phages (Stokar-Avihail et al., 2023). Phage SPO1 mutant gained the capacity to infect non-host *Bacillus* species and phage-resistant bacteria when mutations occurred in two genes encoding the baseplate and tail fibers required for host attachment (Habusha et al., 2019). Similarly, single-nucleotide mutations for tail and baseplate components of Kayvirus led to diverse RBPs in the phage mutant, which allowed the phage mutant to recognize more *Staphylococcus aureus* strains and thus exhibit a broader lysis spectrum (Botka et al., 2019). Besides tail fiber, the point mutations in the receptor-binding tail spike protein of *Shigella* phage Sf6 were also demonstrated to promote the phage host range evolutions (Subramanian et al., 2022). The tail fiber-dependent host specificity generates phage mutants through genetic engineering to rapidly expand the phage host range and overcome bacterial resistance. The highly diverse phage libraries containing phage mutants with altered host ranges that suppress bacterial resistance were created through site-directed mutagenesis of host-range-determining regions (HRDRs) in the T3 phage tail fiber protein (Yehl et al., 2019). The T4-like phage WG01 directly expanded its host range by replacing its HRDR with that of another phage, QL01, compared to the wild-type strains (Chen et al., 2017).

Compared with the frequently reported tail fiber proteins, the tail tubular-dependent host specificity of phages has not been well studied presently. In our study, we identified and demonstrated the adaptive evolution of *Acinetobacter* phage vB_Ab4_Hep4 towards a non-host strain through a point mutation in the tail tubular. The mutations of the tail tubular protein B or the tail fiber protein of *Aquamicrobium* phage P14 increased the phage’s predation efficacy towards *Alcaligenaceae H5* (de Leeuw et al., 2020). Interestingly, in another study, the single nucleotide substitution in the tail tubular genes was confirmed to enhance phage mutants’ thermal stability without affecting their lytic activity compared to wild-type ancestral phages (Kering et al., 2020). The distinct hypervariable mutation locus that correlated with the host range expansion in the tail tubular protein B of Abp4 seems to represent a novel putative host range determinant. The tail tubular mutation of the phage did not alter the type of host receptor. The DNA similarity of capsule biosynthesis gene clusters in Ab4 and Ab170013 was only 69.84%, indicating that the fine structures of their capsules are different. The difference in capsule structure seems to be the reason for the diverse infection situations between the phages and the *A. baumannii* strains in our study. The minor structural alteration of TTPBm caused by a single amino acid substitution could just meet with the recognition for the distinctive CPS of Ab170013, enabling Abp4-M to infect the non-host strain.

In this study, we isolated and characterized a novel lytic phage vB_Ab4_Hep4, which exhibited high lytic activity and significant treatment potential against clinically sourced MDRAB strains. The increasing antibiotic resistance of *A. baumannii* strains has necessitated the isolation and characterization of more novel lytic phages to enrich the phage library and develop phage cocktails therapy. Meanwhile, we identified and demonstrated the host range expansion of the phage driven by a spontaneous tail tubular mutation. Multiple studies have shown that it is possible to expand the host range of phages by gene-modifying tail fiber proteins for better therapeutic efficacy against clinical MDR bacterial infections. The spontaneous tail tubular mutations may be widespread in *Acinetobacter* phages, which can expand the host ranges of phages and allow them to better circumvent resistance during treatments. The phages’ tail tubular-dependent host specificity demonstrated in our study may provide new insights into extending host range by engineering tail tubular proteins.

## Data availability statement

The datasets presented in this study can be found in online repositories. The names of the repository/repositories and accession number(s) can be found below: https://www.ncbi.nlm.nih.gov/genbank/, OP019135; https://www.ncbi.nlm.nih.gov/genbank/, OR075895.

## Author contributions

PH: Data curation, Investigation, Methodology, Visualization, Writing – original draft, Writing – review & editing. FC: Formal analysis, Methodology, Software, Validation, Writing – review & editing. QQ: Data curation, Investigation, Methodology, Writing – original draft. HG: Investigation, Methodology, Visualization, Writing – original draft. XY: Investigation, Methodology, Validation, Writing – original draft. TX: Software, Visualization, Writing – review & editing. RW: Conceptualization, Data curation, Validation, Writing – review & editing. XJ: Conceptualization, Formal analysis, Investigation, Writing – review & editing. ML: Conceptualization, Resources, Writing – review & editing. PZ: Conceptualization, Methodology, Writing – review & editing. GL: Conceptualization, Funding acquisition, Methodology, Writing – review & editing.
