# Supplementary material for: Host range expansion of Acinetobacter phage vB_Ab4_Hep4 driven by a spontaneous tail tubular mutation
Source: Front Cell Infect Microbiol. 2024 Feb 16;14:1301089. doi: 10.3389/fcimb.2024.1301089 (PMC10904470; doi:10.3389/fcimb.2024.1301089)
Supplement: Supplementary file 1 [file DataSheet_1.docx]

**Table S1:** Primers used in this study

| **Gene** | **Primers Name** | **Sequences (5’-3’)** |
| --- | --- | --- |
| *16S rRNA* | 16S-F | AGAGTTTGATCCTGGCTCAG |
|  | 16S-R | GGTTACCTTGTTACGACTT |
| *Tail tubular* | PET42a-tailtuber-BamHI-GST-F | GGATATCGGGGATCCATTCTTGAGGGAGTGTACCC |
|  | PET42a-tailtuber-XhoI-GST-R | GTGGTGGTGCTCGAGTTATAAACGTCTCCGTCCTC |
| *pgm* | Ab4-pgm-F | AATGATGCGGGTGTGAATGT |
|  | Ab4-pgm-R | TAATGTTGCATTGACTGAGC |
| *gpi* | Ab4-gpi-F | CACAGCTTACAGCCTGTGTT |
|  | Ab4-gpi-R | GTGACCCATGCACTATGCGAT |
| *ugd* | Ab4-ugd-F | GAGCATAGCTTGACTCAACA |
|  | Ab4-ugd-R | GCTGTACTACACTGTGGTGT |
| *gtrOC20* | Ab170013-gtrOC20-F | GTGATAAAGATCAGATTGCA |
|  | Ab170013-gtrOC20-R | TATTGTTCCAAGTTATAACC |

**Table S2:** Phage vB_Ab4_Hep4 gene annotations

| **Type** | **Start** | **End** | **Length (bp)** | **Function** | **Functional Classification** |
| --- | --- | --- | --- | --- | --- |
| CDS 1 | 2466 | 49 | 2418 | Phage DNA-directed RNA polymerase | DNA metabolism and replication |
| CDS 2 | 3125 | 2475 | 651 | Phage dNMP kinase | DNA metabolism and replication |
| CDS 3 | 4060 | 3125 | 936 | Phage phosphodiesterase with HTH domain | DNA metabolism and replication |
| CDS 4 | 4504 | 4064 | 441 | Phage endonuclease | DNA metabolism and replication |
| CDS 5 | 5070 | 4501 | 570 | Phage-encoded tRNA nucleotidyltransferase | DNA metabolism and replication |
| CDS 6 | 6040 | 5060 | 981 | Phage exonuclease | DNA metabolism and replication |
| CDS 7 | 6166 | 6047 | 120 | Phage Zn-ribbon domain-containing protein | Structure module |
| CDS 8 | 7113 | 6223 | 891 | Phage protein p21 | Structure module |
| CDS 9 | 7356 | 7117 | 240 | hypothetical protein | Hypothetical protein |
| CDS 10 | 9748 | 7346 | 2403 | Phage DNA-directed DNA polymerase | DNA metabolism and replication |
| CDS 11 | 10119 | 9997 | 123 | Phage-associated ATP-dependent DNA ligase | DNA metabolism and replication |
| CDS 12 | 10975 | 10145 | 831 | Phage-associated ATP-dependent DNA ligase | DNA metabolism and replication |
| CDS 13 | 11706 | 10972 | 735 | Phage-associated ATP-dependent DNA ligase | DNA metabolism and replication |
| CDS 14 | 13007 | 11709 | 1299 | Phage DNA helicase | DNA metabolism and replication |
| CDS 15 | 13256 | 13020 | 237 | Phage protein | Structure module |
| CDS 16 | 13573 | 13256 | 318 | Phage protein | Structure module |
| CDS 17 | 13887 | 13573 | 315 | hypothetical protein | Hypothetical protein |
| CDS 18 | 14690 | 13884 | 807 | Phage primase/helicase protein Gp4A | DNA metabolism and replication |
| CDS 19 | 14889 | 14674 | 216 | Phage DNA-binding protein | DNA packing module |
| CDS 20 | 15097 | 14879 | 219 | Phage DNA-binding protein | DNA packing module |
| CDS 21 | 15282 | 15094 | 189 | Phage protein | Structure module |
| CDS 22 | 15436 | 15269 | 168 | Phage protein | Structure module |
| CDS 23 | 15878 | 15447 | 432 | Phage protein | Structure module |
| CDS 24 | 16366 | 15878 | 489 | Phage protein | Structure module |
| CDS 25 | 16835 | 16437 | 399 | Phage protein | Structure module |
| CDS 26 | 16984 | 16844 | 141 | Hypothetical protein | Hypothetical protein |
| CDS 27 | 17099 | 16995 | 105 | Hypothetical protein | Hypothetical protein |
| CDS 28 | 17692 | 17096 | 597 | Hypothetical protein | Hypothetical protein |
| CDS 29 | 18001 | 17771 | 231 | Hypothetical protein | Hypothetical protein |
| CDS 30 | 18101 | 17988 | 114 | putative membrane protein | Structure module |
| CDS 31 | 18466 | 18092 | 375 | Hypothetical protein | Hypothetical protein |
| CDS 32 | 18977 | 18468 | 510 | Phage protein p03 | Structure module |
| CDS 33 | 19301 | 19050 | 252 | Hypothetical protein | Hypothetical protein |
| CDS 34 | 20010 | 19732 | 279 | Hypothetical protein | Hypothetical protein |
| CDS 35 | 21295 | 20963 | 333 | Hypothetical protein | Hypothetical protein |
| CDS 36 | 21508 | 21305 | 204 | Phage protein | Structure module |
| CDS 37 | 21602 | 21468 | 135 | Phage DNA-binding protein | DNA packing module |
| CDS 38 | 23536 | 21599 | 1938 | Phage terminase large subunit Gp19 | DNA packing module |
| CDS 39 | 23854 | 23546 | 309 | Phage terminase small subunit Gp18 | DNA packing module |
| CDS 40 | 23965 | 23864 | 102 | Phage endolysin | Lysis module |
| CDS 41 | 24564 | 24007 | 558 | Phage endolysin | Lysis module |
| CDS 42 | 24886 | 24551 | 336 | Phage holin | Lysis module |
| CDS 43 | 27016 | 24896 | 2121 | Phage non-contractile tail fiber protein Gp17 | Structure module |
| CDS 44 | 30121 | 27023 | 3099 | internal virion protein C | Lysis module |
| CDS 45 | 33016 | 30131 | 2886 | Phage internal virion protein B | Lysis module |
| CDS 46 | 33703 | 33029 | 675 | putative internal virion protein B | Lysis module |
| CDS 47 | 35994 | 33703 | 2292 | Phage non-contractile tail tubular protein Gp12 | Structure module |
| CDS 48 | 36629 | 36003 | 627 | Phage non-contractile tail tubular protein Gp11 | Structure module |
| CDS 49 | 37089 | 36796 | 294 | Phage tail needle protein | Structure module |
| CDS 50 | 37286 | 37101 | 186 | Hypothetical protein | Hypothetical protein |
| CDS 51 | 38374 | 37343 | 1032 | Phage major capsid protein Gp10A | Structure module |
| CDS 52 | 39250 | 38390 | 861 | Phage capsid assembly scaffolding protein p31 | Structure module |
| CDS 53 | 40815 | 39259 | 1557 | Phage collar, head-to-tail connector protein Gp8 | Structure module |
| CDS 54 | 41075 | 40824 | 252 | Phage structural protein p29 | Structure module |
| CDS 55 | 41269 | 41072 | 198 | Phage protein | Structure module |
| CDS 56 | 41462 | 41385 | 78 | Phage DNA-directed RNA polymerase | DNA metabolism and replication |

**Table S3:** Phages used for the phylogenetic analysis

| **Phage** | **Accession number** | **Reference** |
| --- | --- | --- |
| *Acinetobacter* phage vB_Ab4_Hep4 | OP019135 | — |
| *Acinetobacter* phage AB3 | NC_021337 | (Zhang et al., 2015) |
| *Acinetobacter* phage AbKT21phiIII | NC_048142 | (Nir-Paz et al., 2019) |
| *Acinetobacter* phage AbTP3phi1 | OL770263 | — |
| *Acinetobacter* phage vB_AbaP_ZHSHW | OM925528 | — |
| *Acinetobacter* phage vB_AbaP_EPab_B | OQ730212 | — |
| *Acinetobacter* phage vB_AbaP_APK116 | MN807295 | — |
| *Acinetobacter* phage Fri1 | NC_028848 | — |
| *Acinetobacter* phage vB_AbaP_AS11 | NC_041915 | (Popova et al., 2017) |
| *Acinetobacter* phage vB_AbaP_PE21 | OL964948 | — |
| *Acinetobacter* phage Abgy2021-4-1 | OR770645 | — |
| *Acinetobacter* phage Abp1 | NC_021316 | (Huang et al., 2013) |
| *Acinetobacter* phage SWH-Ab-3 | NC_047883 | — |
| *Acinetobacter* phage vB_AbaP_APK32 | MK257722 | — |
| *Acinetobacter* phage phiAB6 | KT339321 | (Lai et al., 2016) |
| *Acinetobacter* phage Abgy2021-6-2 | OR770644 | — |

Note: “—” indicates the phage has not been published.


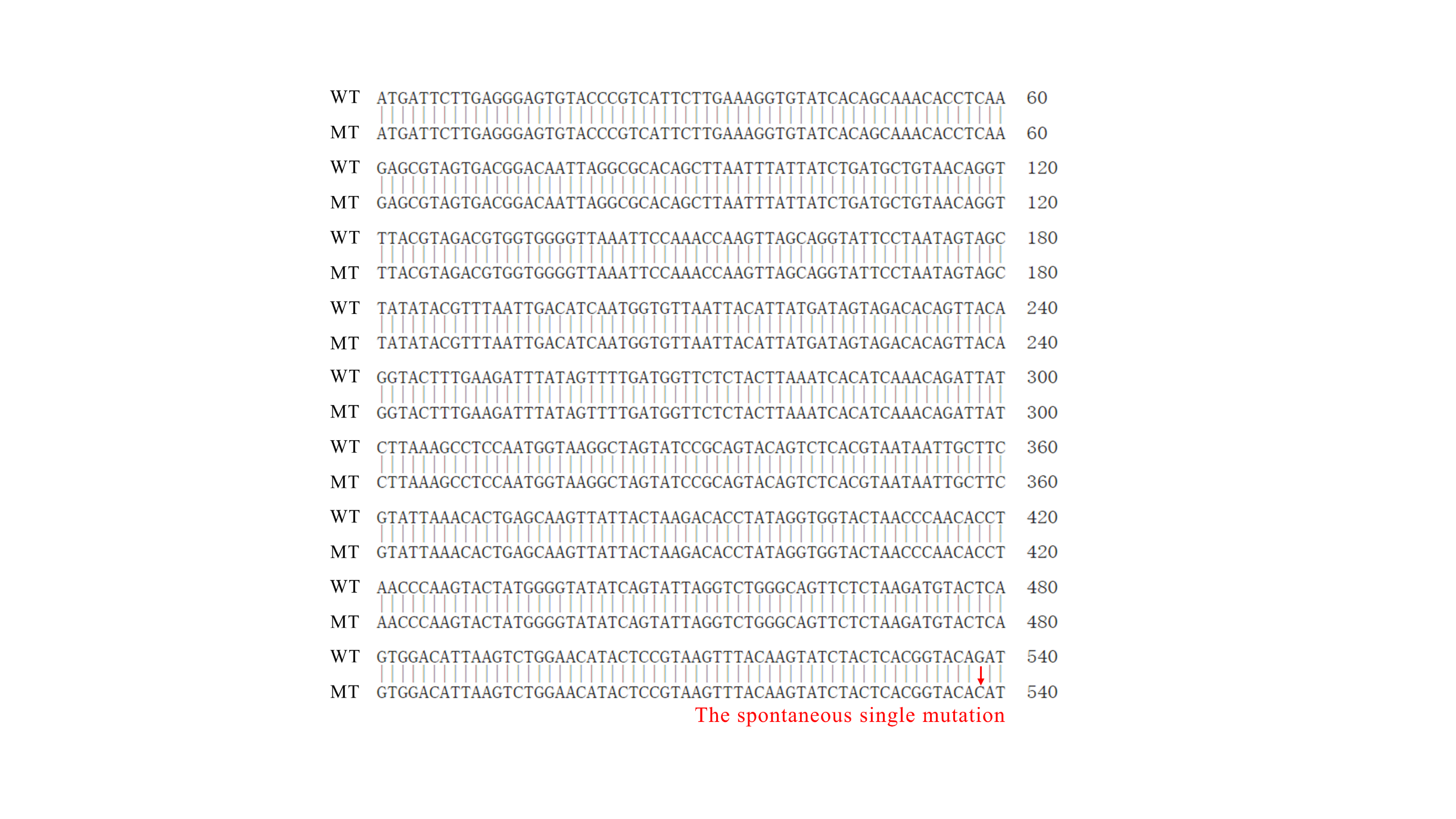


**Fig. S1:** Information about the mutation site of phage mutant vB_Ab4_Hep4-M (Abp4-M)

**
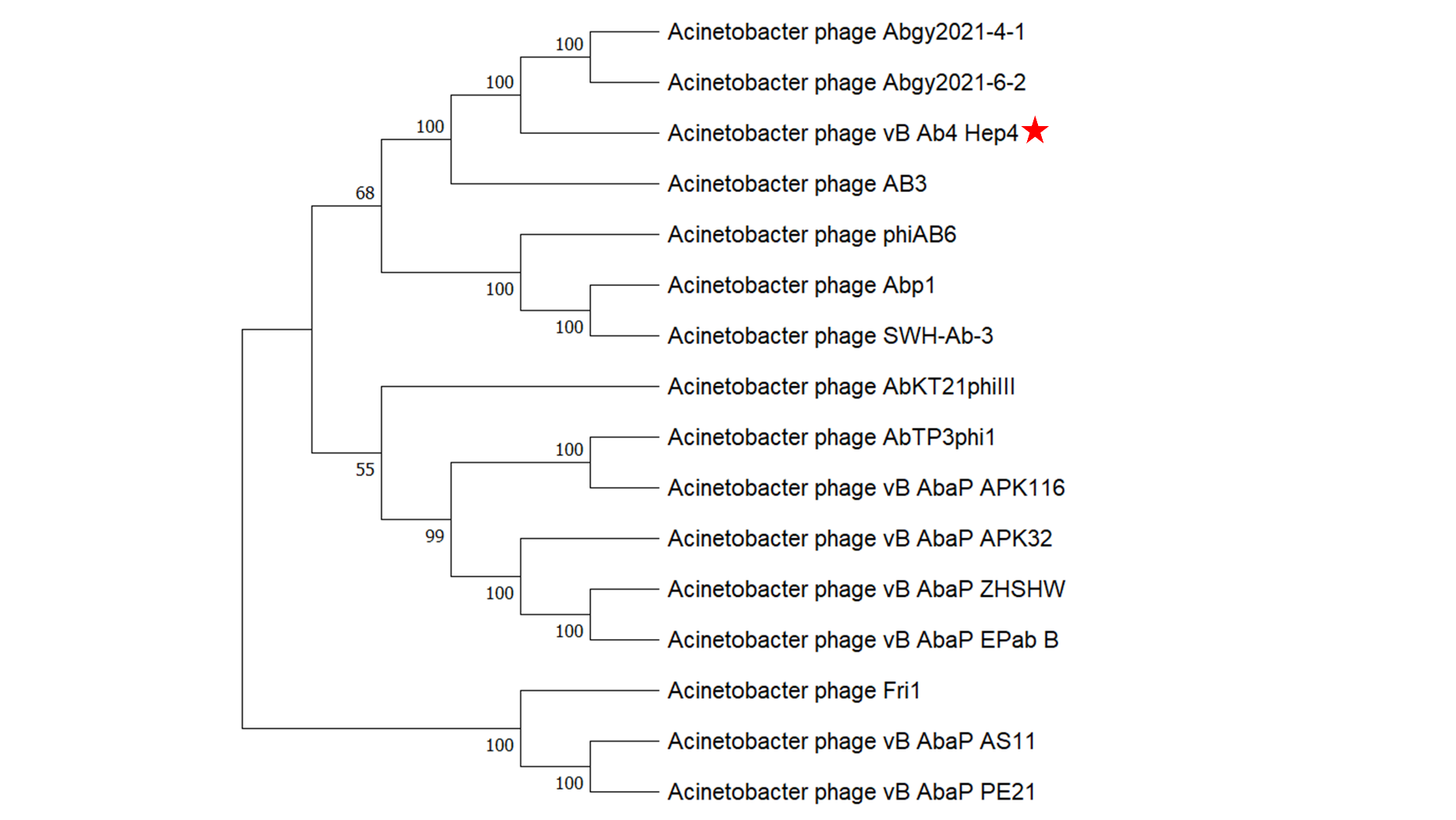
**

**Fig. S2:** The bootstrap consensus tree of phylogenetic analyses of relatedness between Abp4 and other *Acinetobacter* phages based on whole genome sequences using MEGA_11.0 software.


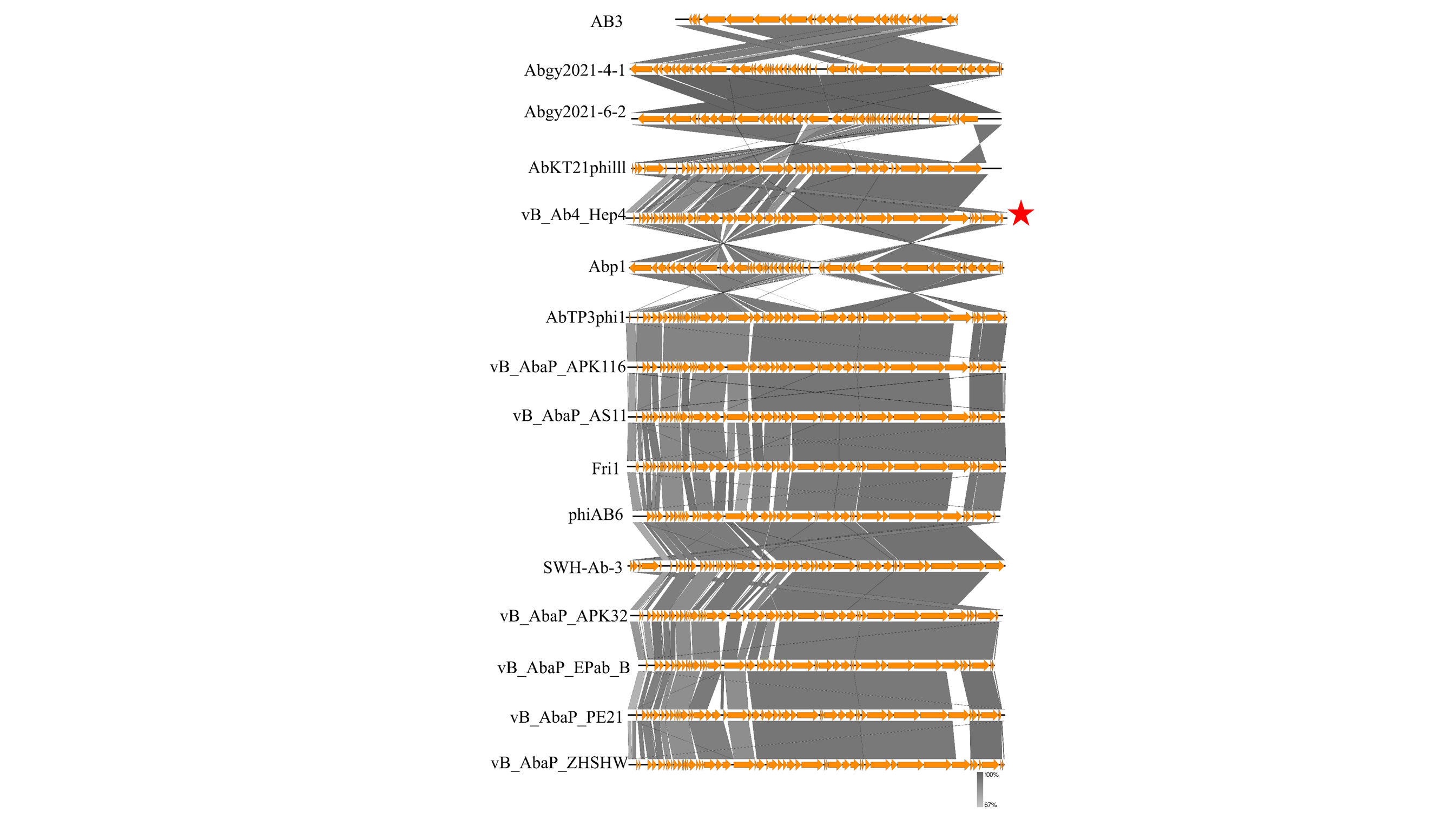


Fig. S3: The comparative genomic graph among all phages used for the phylogenetic analyses.

**References**

Huang, G., Le, S., Peng, Y., Zhao, Y., Yin, S., Zhang, L., et al. (2013). Characterization and genome sequencing of phage Abp1, a new phiKMV-like virus infecting multidrug-resistant Acinetobacter baumannii. Curr Microbiol 66(6), 535-543. doi: 10.1007/s00284-013-0308-7.

Lai, M.J., Chang, K.C., Huang, S.W., Luo, C.H., Chiou, P.Y., Wu, C.C., et al. (2016). The Tail Associated Protein of Acinetobacter baumannii Phage ΦAB6 Is the Host Specificity Determinant Possessing Exopolysaccharide Depolymerase Activity. PLoS One 11(4), e0153361. doi: 10.1371/journal.pone.0153361.

Nir-Paz, R., Gelman, D., Khouri, A., Sisson, B.M., Fackler, J., Alkalay-Oren, S., et al. (2019). Successful Treatment of Antibiotic-resistant, Poly-microbial Bone Infection With Bacteriophages and Antibiotics Combination. Clin Infect Dis 69(11), 2015-2018. doi: 10.1093/cid/ciz222.

Popova, A.V., Lavysh, D.G., Klimuk, E.I., Edelstein, M.V., Bogun, A.G., Shneider, M.M., et al. (2017). Novel Fri1-like Viruses Infecting Acinetobacter baumannii-vB_AbaP_AS11 and vB_AbaP_AS12-Characterization, Comparative Genomic Analysis, and Host-Recognition Strategy. Viruses 9(7). doi: 10.3390/v9070188.

Zhang, J., Liu, X., and Li, X.J. (2015). Bioinformatic analysis of phage AB3, a phiKMV-like virus infecting Acinetobacter baumannii. Genet Mol Res 14(1), 190-198. doi: 10.4238/2015.January.16.2.
